# Problem drinking as a risk factor for tuberculosis: a propensity score matched analysis of a national survey

**DOI:** 10.1186/1471-2458-13-871

**Published:** 2013-09-22

**Authors:** Annibale Cois, Rodney Ehrlich

**Affiliations:** 1 School of Public Health and Family Medicine, University of Cape Town, Anzio Road, Observatory 7925, Cape Town, South Africa

**Keywords:** Tuberculosis, Alcohol, Problem drinking, Multiple confounders, Propensity score, Sensitivity analysis

## Abstract

**Background:**

Epidemiological and other evidence strongly supports the hypothesis that problem drinking is causally related to the incidence of active tuberculosis and the worsening of the disease course. The presence of a large number of potential confounders, however, complicates the assessment of the actual size of this causal effect, leaving room for a substantial amount of bias. This study aims to contribute to the understanding of the role of confounding in the observed association between problem drinking and tuberculosis, assessing the effect of the adjustment for a relatively large number of potential confounders on the estimated prevalence odds ratio of tuberculosis among problem drinkers vs. moderate drinkers/abstainers in a cross-sectional, nationally representative sample of the South African adult population.

**Methods:**

A propensity score approach was used to match each problem drinker in the sample with a subset of moderate drinkers/abstainers with similar characteristics in respect to a set of potential confounders. The prevalence odds ratio of tuberculosis between the matched groups was then calculated using conditional logistic regression. Sensitivity analyses were conducted to assess the robustness of the results in respect to misspecification of the model.

**Results:**

The prevalence odds ratio of tuberculosis between problem drinkers and moderate drinkers/abstainers was 1.97 (95% CI: 1.40 to 2.77), and the result was robust with respect to the matching procedure as well as to incorrect adjustment for potential mediators and to the possible presence of unmeasured confounders. Sub-population analysis did not provide noteworthy evidence for the presence of interaction between problem drinking and the observed confounders.

**Conclusion:**

In a cross-sectional national survey of the adult population of a middle income country with high tuberculosis burden, problem drinking was associated with a two fold increase in the odds of past TB diagnosis after controlling for a large number of socio-economic and biological confounders. Within the limitations of a cross-sectional study design with self-reported tuberculosis status, these results adds to previous evidence of a causal link between problem drinking and tuberculosis, and suggest that the observed higher prevalence of tuberculosis among problem drinkers commonly found in population studies cannot be attributed to the confounding effect of the uneven distribution of other risk factors.

## Background

Substantial evidence supports the hypothesis that heavy drinking patterns and/or alcohol use disorders (problem drinking), but not moderate consumption, are causally related to the incidence of active tuberculosis (TB) and the worsening of the disease course [1]. Epidemiological studies have long observed that compared to the general population problem drinking is more frequent among TB patients, and, similarly, that the prevalence of TB is higher among patients with alcohol-related disorders. Controlled studies have consistently confirmed this association, and a recent meta-analysis of case-control and cohort studies indicated a substantial increase in the TB risk among people who drank more than 40 g alcohol per day, and/or had a clinical diagnosis of alcohol use disorder, with a pooled risk ratio of 2.94 (95% CI: 1.89–4.59) [2]. A subsequent systematic review of published articles on risk factors associated with recent transmission of TB reported comparable results, providing a pooled risk ratio of 2.27 (95% CI: 1.69–3.06) for subjects with a history of excessive alcohol consumption [3]. There is also biological and sociological plausibility for a causal relationship between alcohol exposure and incidence of TB via impairment of the immune system, specific social mixing patterns among people with heavy alcohol use, and social marginalization and drift [1,2].

Despite the sound evidence that problem drinking increases the risk of TB infection and progression to disease, the presence of a large number of potential confounders complicates the assessment of the actual size of this causal effect, leaving room for a substantial amount of bias. This may partly explain the heterogeneity of results across the different studies [2]. Some important confounding factors — e.g. socioeconomic status — are difficult to measure and fully control for. Malnutrition, diabetes and indoor air pollution caused by burning of solid fuels are putative risk factors for TB [4-6] and are often associated with alcohol use, but have been rarely considered as possible confounders in epidemiological studies on the association between alcohol and TB. On the other hand, adjustment for factors partially lying in the causal pathway between problem drinking and TB, such as malnutrition and socieconomic status, could also bias the observed effect, most likely downwards.

This study aims to contribute to the understanding of the role of confounding in the observed association between problem drinking and TB. Specifically, it assesses the effect of the adjustment for a relatively large number of potential confounders on the estimated prevalence odds ratio (POR) of TB disease among problem drinkers vs. moderate drinkers/abstainers in a cross-sectional, nationally representative sample of the South African adult population. Possible interaction between alcohol and other risk factors are explored through restriction of the investigation to relevant sub-groups, and a sensitivity analysis is conducted to estimate the robustness of the calculated POR with respect to improper adjustment for potential mediators, to the presence of unmeasured confounders and to the choices made in the statistical modelling.

## Methods

### Participants

This study used data from the 2003 South African Demographic and Health Survey (SADHS), a cross-sectional study based on an international methodology and aimed at providing data on population, health and nutrition in developing countries. Ethical approval for the conduct of the SADHS was granted by the Ethics Committee of the South African Medical Research Council (MRC). A copy of the full dataset, with all identifying information removed, was provided by the MRC, with permission to use it for the secondary analyses presented in this article. The survey collected a broad range of information on participants, including sociodemographic characteristics, past TB diagnoses, pattern of alcohol use, anthropometric measurements, eating habits and known bio-behavioural risk factors for TB infection and progression. The SADHS utilised a nationally representative sample of the South African population, stratified by province and by type of residence (urban or rural). A two stage sampling strategy was implemented, with census enumeration areas as primary sampling units, and secondary units represented by households [7]. The data analysed here refer to the 8 115 respondents of the adult questionnaire, for which the eligible population was constituted by all adults aged 15 years and more present in every second household selected for the survey.

The overall response rate for the SADHS was 71% at individual level, with large differences between urban and rural areas (65% and 82%, respectively), with the main reason of nonresponse being refusal [7].

### Measures

#### Sociodemographic variables

Various sociodemographic characteristics of participants were considered: age, gender, race, education, rural or urban residence, wealth, access to medical insurance and average number of people per room in the household. Age was categorised in six groups to capture the known bimodal distribution of TB prevalence by age in the South African population, with a peak in early adulthood and another among elders [8]. Race, enduringly correlated with socioeconomic status in South Africa, was self-defined by participants according to the historical “population group” categorization used in South Africa (Black/African, Asian/Indian, Coloured, White). Education was measured in years of completed schooling, and categorised in four classes. Using principal component analysis, a *wealth index* was calculated by the South African Medical Research Council from information regarding household assets in the SADHS household questionnaire. Respondents were classified in quintiles of this index, with higher ranking representing greater household wealth [9]. If the average reported number of people per room was > 3 subjects, respondents were considered as belonging to an overcrowded household.

#### TB disease

A positive response to the question “*Has a doctor or nurse or health worker at a clinic or hospital told you that you have or have had TB?*” was considered as a proxy for TB disease.

#### Problem drinking

Problem drinking was assessed using the CAGE questionnaire in its original form with no time constraints, in which questions refer to the subjects’ lifetime experience [10]. The CAGE questionnaire is one of the most frequently used screening tools for alcohol problems both in clinical practice and for research purpose. Its predictive validity in various non-clinical populations is well supported [11,12], and specifically confirmed in a validation study conducted in South Africa [13]. In accordance with the prevalent literature, an affirmative response to ≥ 2 of the four questions of the CAGE questionnaire was regarded as a proxy for problem drinking. Subjects with any lifetime use of alcohol but less than two affirmative responses were considered as moderate drinkers.

#### Exposure to smoke

Regarding the use of tobacco products, subjects were classified in three categories: never used, ever regular smoking, ever used smokeless tobacco. Domestic air pollution was assessed by asking the respondents to select from a list the type of fuel used for cooking, lighting and heating, and classifying them as exposed to *smoky fuel* for any use of coal, candles, firewood and animal dung. A binary variable was created to represent occupational exposures from the following survey question: “*Have you ever worked in a job where you were regularly exposed to smoke, dust, fumes or strong smells?*”.

#### Macro and micro nutrition

BMI was used as a measure of macro nutrition in this study. Measures of weight and height were recorded in the SADHS dataset by trained fieldworkers. Excluding measures with implausible values, BMI in *kg*/*m*^2^ was calculated from these values and subjects were categorised as normal weight, underweight, overweight or obese according to the World Health Organization’s cut-offs [14]. A 30 item food frequency questionnaire was used to estimate the average intake of 13 micronutrients, which was compared with the age and sex specific Recommended Daily Allowance (RDA) reference values. Respondents were classified in four categories, from adequate micronutrient intake to severe deficiency ( [7], p.402).

#### Diabetes

Subjects who responded affirmatively to the question “*Has a doctor or nurse or health worker at a clinic or hospital told you that you have or have had diabetes?*” and/or who were on anti-diabetic medication were classified as diabetic.

### Statistical analysis

Analyses were carried out using Stata^®;^ Statistical Software Version 12 for Windows [15]. When appropriate, statistical models and point estimates were adjusted for the multi-stage, stratified sampling scheme of the SADHS using design details and post-stratification sampling weights provided by the Medical Research Council [7]. Confidence intervals for the estimated parameters were calculated through bootstrapping with 750 replications [16].

#### Association between problem alcohol use and TB disease

The POR representing the association between problem alcohol use and TB, with basic adjustment for age and gender, was estimated by means of logistic regression.

A propensity score (PS) approach was applied in the estimation of the association taking into account the whole set of potential confounders [17]. According to this approach, the PS — i.e. the conditional probability of being a problem drinker given the values of all potential confounders — was estimated for each subject and used to create comparable groups of problem users (exposed) and moderate drinkers/abstainers (unexposed). It can be shown that, *in absence of unmeasured confounding factors*, two subjects having the same PS but different exposure status can be considered as if they were randomly assigned to the exposure. Thus, matching on the PS can balance the distribution of the observed confounders and remove bias that may arise due to them [18].

Compared to traditional multivariate modelling, PS analyses have usually greater requirements in respect of minimum sample size and are analytically more complex [19]. Moreover, when the outcome of interest is not rare and the number of potential confounders is moderate, they tend to provide results often indistinguishable from those provided by traditional multivariate techniques [20]. The results may be even more biased than those provided by other methods when, in presence of relatively strong unmeasured confounders, variables associated with the exposure but not associated with the outcome are erroneously included in the model used to estimate the PS [21,22].

However, it is increasingly acknowledged that PS analysis offers substantial advantages over traditional multivariate modelling when the outcome is rare relative to the number of confounders, provided that the number of study subjects in the smaller exposure group is sufficiently large to warrant reliable PS estimation [20]. In this case logistic regression techniques perform poorly and the results of a recent simulation study show that PS estimates are consistently less biased, more precise and less sensitive to errors in the estimation of the effect of the confounders on the outcome when the number of events per confounder is ≤7 [23]. As a further benefit, PS models allow for the independent assessment of the balance of the observed covariates between exposed and unexposed, which is not possible with traditional multivariate regression modelling, in which the relationships between outcome, exposure and confounders are estimated jointly [18].

In this study, PS was estimated for each subject by means of a probit regression model including all available potential confounders and, as further covariates, population strata and a categorical version of the sampling weights. The estimated score was then used to match each problem drinker in the sample with four unexposed individuals (nearest neighbourhood matching on the odds of the PS, with repetition, caliper  = 0.01) [18,19], and the comparability of the two groups was evaluated by the successful balance of the observed covariates. Finally, the POR of TB among problem drinkers vs. moderate drinkers/abstainers was calculated using conditional logistic regression in the matched groups.

#### Interaction between problem alcohol use and confounders

The above procedure was repeated for a variety of sub-populations characterised by constant values of different confounders. PORs and confidence intervals were calculated and compared, to verify the extent to which the association between problem drinking and TB was homogeneous across groups.

#### Sensitivity analyses

Three types of sensitivity analysis were performed. First, we assessed the robustness of the estimates with respect to the accuracy of the matching procedure and the ratio exposed/unexposed. Secondly, the models were refitted excluding variables potentially belonging to the causal pathway between problem drinking and TB disease, in order to assess the possibility of improper adjustment for partial mediators. Finally, adapting the procedure suggested by Ichino *at al.*[24], we randomly generated a large set of hypothetical confounders and estimated the percentage reduction on the observed POR between TB disease and problem drinking when each of them was introduced in the model. The results of the repeated simulations were then jointly depicted in a contour plot, whit the axes representing the degree of association of the simulated confounders with the exposure and the outcome. This allowed us to visually identify lower bounds for the strength of the association that potential unmeasured confounders should have with the outcome and the exposure to offset the observed POR or to reduce its value below any specific threshold.

Details of statistical procedures and further references are reported in Additional file 1.

## Results

Overall, the number of missing values in the dataset was relatively low, between 0 for basic demographic characteristics and 3.7% of the total sample size for BMI and problem drinking. This made it feasible to adopt a complete-case approach to the analyses, thus discarding observations with missing values in the estimation of PS and POR. Recent simulation studies have shown that this approach can provide valid inferences compared to more complex and computationally demanding imputation procedures, where higher statistical efficiency often comes at the cost of a greater amount of bias in the estimates of interest [25].

The total number of subjects reporting past diagnoses of TB was 205 (2.5% of the total sample), with a higher prevalence among males (3.1%) than among females (2.1%). Considering the pattern of missing data and the use of dummies to model categorical variables, this low number of positive outcomes lead to 5.5 events per covariate, well below the threshold which makes multivariate logistic modelling advisable.

Past or present problem drinking was quite common among males (24.8%), but much less so among females (8.5%). TB prevalence was more than 3 times higher among problem drinkers than among moderate drinkers/abstainers (6.0% vs. 1.9%).

Additional sample characteristics are shown in Table 1.

**Table 1:** Characteristics of the 2003 South African demographic and health survey adult sample

**Variable**	**N**	**Percentage**	**Frequency**
TB	8 053	2.5%	205
Problem drinking	7 811	15.2%	1 185
Age (*years*)	8 115		
15–24		29.3%	2 376
25–34		20.5%	1 667
35–44		17.5%	1 418
45–54		14.6%	1 185
55–64		9.8%	793
>64		8.3%	676
Men	8 115	41.0%	3 328
Race	8 106		
Black		75.0%	6 081
Coloured		12.3%	995
White		8.8%	717
Asian		3.7%	297
Other		0.2%	16
Education	8 076		
None		12.6%	1 017
Primary		15.3%	1 234
Secondary		64.9%	5 238
Tertiary		7.3%	587
Urban residence	8 115	57.2%	4 641
Medical insurance	8 092	13.6%	1 101
Overcrowding	8 043	5.9%	473
Tobacco use	8 104		
Never		64.2%	5 202
Ever smoked		28.5%	2 306
Ever smokeless		7.3%	596
Smoky fuel	8 054	36.4%	2 930
Occupational exposure to smoke	8 075	17.9%	1 449
Body mass index	7 813		
Normal		49.6%	3 876
Underweight		7.8%	612
Overweight		23.9%	1 871
Obese		18.6%	1 454
Micronutrient deficiency	8 069		
0		3.0%	245
1		22.4%	1 810
2		39.2%	3 165
3		35.3%	2 849
Diabetes	8 045	4.0%	322

N = number of nonmissing cases.

See text for variables definitions.

Taking into account the sampling scheme, the estimated prevalence of past TB diagnosis in the South African adult population was 2.2% among women (95% CI: 1.7% to 2.8%) and 3.5% among men (95% CI: 2.7% to 4.3%). Problem drinking affected 7.1% of women (95% CI: 6.1% to 8.2%) and 22.1% of men (95% CI: 20.1% to 24.1%). The estimated prevalence of TB was 7.3% (95% CI: 5.4% to 9.1%) among problem drinkers and 2.0% (95% CI: 1.6% to 2.5%) among the unexposed.

### Problem alcohol use and TB

A logistic regression model with adjustment for gender and age alone showed sizably higher odds of past TB among problem drinkers than among other subjects (POR = 3.30; 95% CI: 2.26 to 4.81).

**Figure 1:**
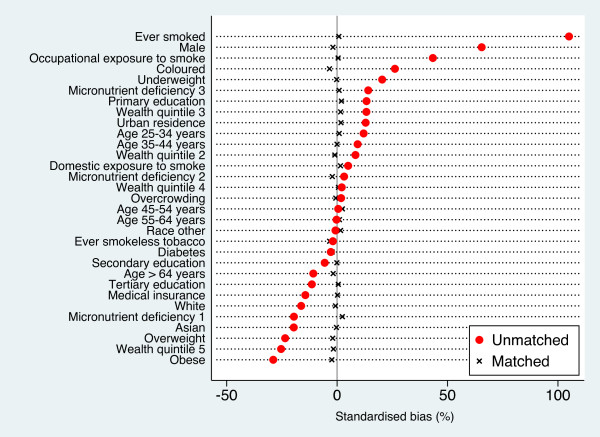
**Standardised percent bias in the distribution of potential confounders of the association between problem drinking and TB in the South-African adult population.** Pre- and post-matching differences in the prevalence of each potential confounder between problem drinkers and moderate drinkers/abstainers as a percentage of the square root of the average of their variances. For definition of variables, see text.

**Figure 2:**
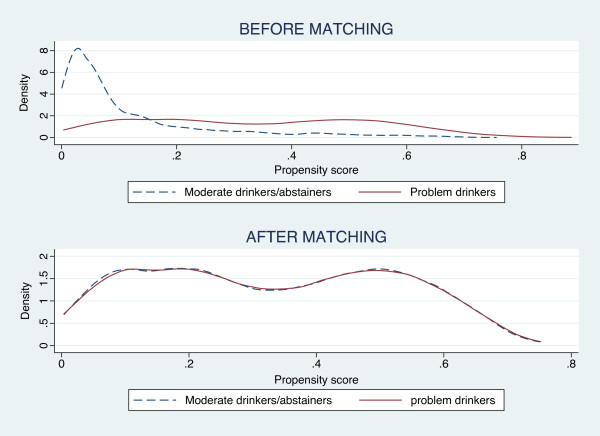
**Estimated propensity of being a problem drinker in the sample, by group.** Pre- and post-matching density estimate of propensity score among problem drinkers and moderate drinkers/abstainers (Epanechnikov kernel, “optimal” bandwidth [26]).

The matching procedure was effective in creating a group of unexposed subjects *comparable* to the group of problem drinkers in respect of all observed confounders. Before matching, the distribution of potential confounders was very different between exposed and unexposed, while the matched groups showed only a slight residual imbalance, with values of standardised bias ≤ 3.5*%* across all variables, down from the pre-matching maximum of 105% (Figure 1). None of the post-matching differences between groups were statistically significant (t-test for difference in means, *p* > 0.4). Distribution of PS were also almost completely overlapped after matching (Figure 2).

The POR of TB disease in problem drinkers relative to matched controls was 1.97 (95% CI: 1.40 to 2.77). As expected, it was lower than the POR adjusted for age and sex alone, but confirmed that problem drinking is associated with doubled odds of TB.

The repetition of the analyses comparing problem drinkers separately with non drinkers and moderate drinkers produced similar results, with modest differences in the adjusted PORs: 2.25 (95% CI: 1.59% to 3.20%) and 1.80 (95% CI: 1.25% to 2.58%), respectively.

### Interaction

**Figure 3:**
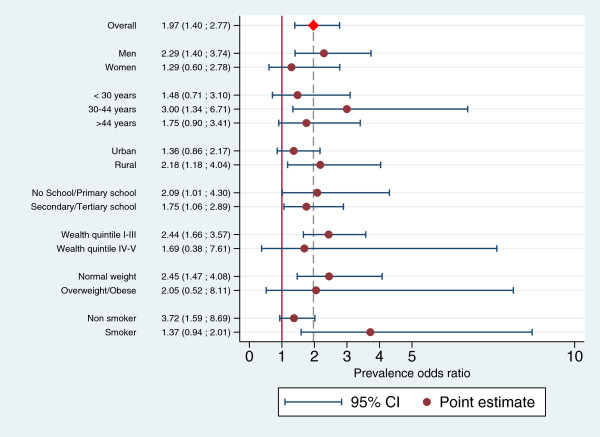
Prevalence odds ratio of TB in problem drinkers vs. moderate drinkers/abstainers and bootstrapped confidence intervals in selected sub-populations.

Figure 3 shows the POR estimates (and relative confidence intervals) for selected sub-populations. Point estimates in subgroups range between 1.29 and 3.73 and their confidence interval largely overlap.

The formal comparison of effect sizes between sub-populations defined by gender, age class, residence, education, wealth quintile and BMI did not provide enough evidence to reject the hypothesis of no difference. Estimated PORs were consistent in direction and all 95% CIs for their ratios included the null value, suggesting that it is unlikely that the association between problem drinking and TB was substantively different across the considered sub-populations.

An exception might be smoking status, where effect sizes (paradoxically 3.72 among non smokers compared to 1.36 among smokers) differed remarkably, and the confidence interval for their ratio did not include the null value (POR_*nonsmokers*_/POR_*smokers*_ = 2.71; 95% CI: 1.07 to 6.87). However, the low precision of the estimate and the lower bound of the confidence interval barely higher than the null value make it difficult to draw any reliable inference about the existence of a true difference by smoking status in the association between problem drinking and TB, especially given that there is no plausible mechanism for an interaction in this direction.

### Unmeasured confounders and sensitivity analysis

The estimated POR was robust in respect to the arbitrary choices in the matching procedure. Changing the matching ratio, increasing its accuracy (by using a ten times narrower caliper) and/or imposing a 1:1 matching without replication produced changes in the point estimate lower than ±15%.

The results of the analyses for possible mediators improperly included as confounders are summarised in Table 2.

**Table 2:** Prevalence odds ratio of TB in problem drinkers vs. moderate drinkers/abstainers and bootstrapped confidence intervals excluding adjustment for selected covariates

**Excluded covariates**	**POR**	**95% CI**
**Fully adjusted**	1.97	1.40–2.77
BMI	1.77	1.28–2.44
Education	2.12	1.43–3.17
Wealth quintile	1.54	1.09– 2.18
Micronutrient deficiency	1.81	1.24–2.65
Education, wealth quintile	2.38	1.67–3.40
Education, wealth quintile, micronutrient deficiency	1.74	1.24–2.45

POR = Prevalence Odds Ratio. CI = Confidence Interval.

Drawing from previous suggestions [1], we considered the possibility that problem drinking leads to social downward drift, thus affecting TB risk via malnutrition and/or unfavourable living conditions. Consequently, we estimated the POR without adjusting for education and/or wealth quintile (as proxies of socioeconomic status), and BMI/micronutrient intake (as proxies of malnutrition). Overall, results did not offer support to the above hypothesis. The *decrease* in the estimated POR excluding adjustment for BMI and/or micronutrient intake is inconsistent with a mediation hypothesis, while the modest increase associated with the exclusion of education does not accord with the decrease observed when neglecting wealth quintile, thus offering very weak support (if any) for the possibility of a mediation path including socieconomic status.

**Figure 4:**
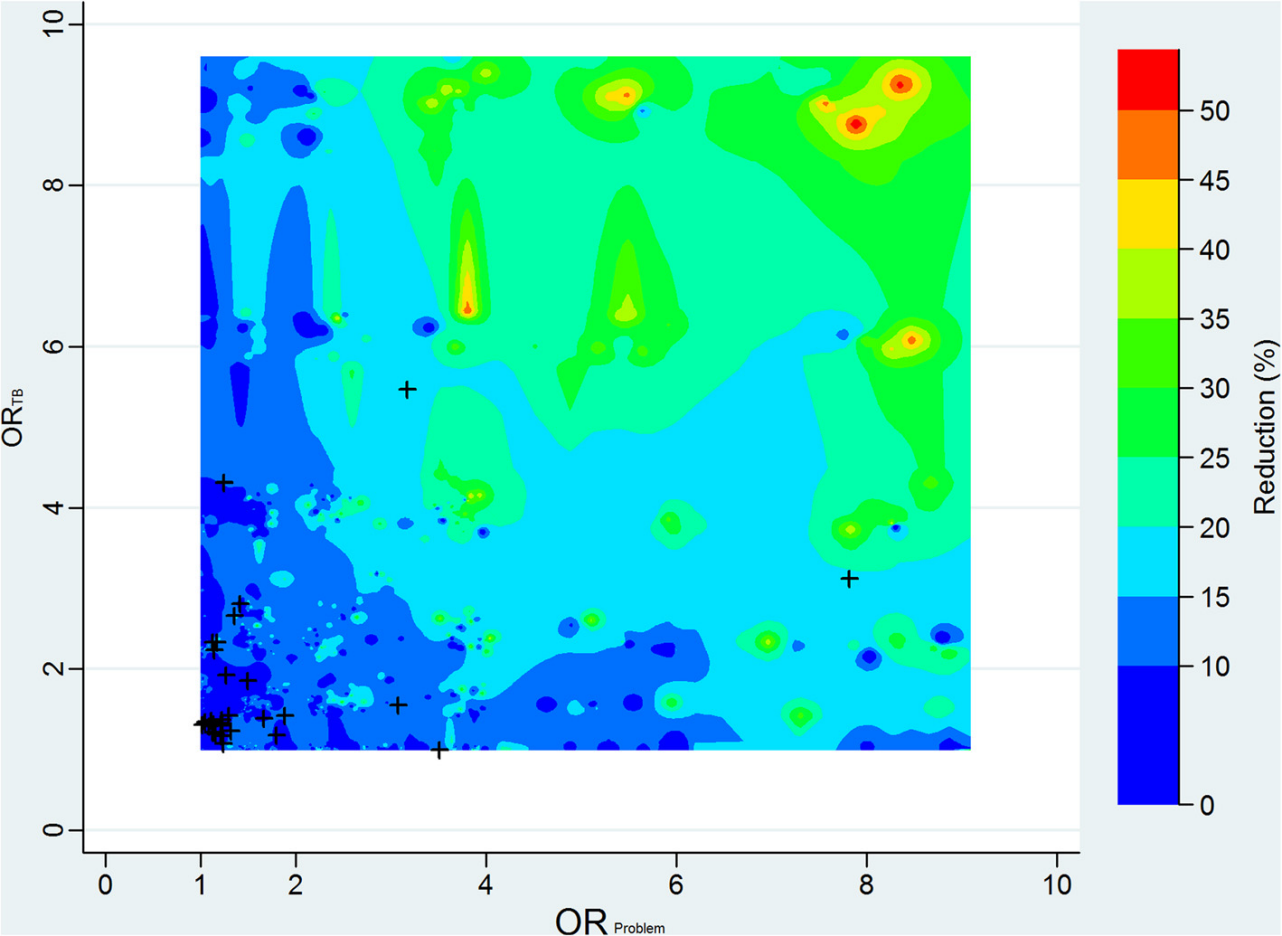
**Sensitivity of the estimated prevalence odds ratio of TB to unmeasured confounders.** Estimated percent reduction of the prevalence odds ratio of TB in problem drinkers vs. moderate drinkers/abstainers when hypothetical, randomly generated, binary confounders are introduced in the models. Axes represent the adjusted odds ratio of the association — irrespective of the direction — of the hypothetical confounder with problem drinking (OR_*Problem*_) and TB (OR_*TB*_). Colours represent couples of values for OR_*Problem*_ and OR_*TB*_ producing the same reduction in the prevalence odds ratio. (N = 1000 simulated confounders. Contour plot, Shepard interpolation [27]).

Figure 4, finally, shows the results of the simulation supporting the robustness of the above analyses with respect to violation of the basic assumption, *per se* untestable, of absence of unmeasured confounding factors (known in PS practice as the assumption of *strong ignorability of treatment assignment*).

Within the limitations of a *brute force* method relying on the random generation of a relatively large — but obviously not exhaustive — number of possible binary confounders, the figure suggests that only adjustment for hypothetical unmeasured factors with an extremely strong association with both TB and problem drinking (ORs > 6) could reduce the POR by about 50% or more, thus accounting for all the observed effect of problem drinking on TB. Even confounders characterised by ORs of 4 could account only for about 35% of the observed effect. By way of comparison, all the observed confounders belonged to the *safe* area of the graph (potential bias < 25%).

## Discussion

The results of our analyses in this representative sample of the South African adult population adds to previous evidence that problem drinking is associated with a substantial increase in TB risk. The comparison between unadjusted and fully adjusted PORs shows that confounding by socioeconomic and bio-behavioural factors plays some role in producing the observed association, but does not explain the full relationship even when the possibility of further, unmeasured, confounders is considered. The sensitivity analysis indicates, in fact, that a potential unobserved binary confounder would have to have had an extremely strong association (ORs > 6) with *both* problem drinking and TB to offset the observed association between these variables. This seems to rule out the possibility that neglecting HIV status in the analysis — an important potential confounder highly prevalent in the South African population and unmeasured in the SADHS — could have substantially biased the estimated POR.

In fact, even if associations between HIV and TB of this strength are realistic [28], this is not true of the relationship between problem drinking and HIV, for which ORs > 2 have rarely been observed, and only in certain high risk populations [29].

Therefore — within the limits of a cross-sectional analysis (see below) — the data are compatible with the hypothesis of a causal effect of problem drinking on TB disease occurrence. Plausible mechanisms suggested in literature are impairment of the immune system, social mixing patterns and social drift. Among these, the study allowed only a partial check of the consistency of the data with the last of these, with no noteworthy support for this hypothesis.

The study adds to the current literature of problem drinking as a risk factor for tuberculosis in several ways.

First, compared to the great majority of studies — which controlled, through design and/or analysis, only for a limited number of confounders — the results of this study take simultaneously into account a large number of factors potentially able to bias the observed association between problem drinking and TB. In particular, the analyses were adjusted for factors rarely considered in literature, i.e. macro- and micro-nutritional status, diabetes, and exposure to air pollution, both domestic and occupational. Moreover, the robustness of the findings has been checked against potential unmeasured confounders and incorrect adjustment for possible mediators.

Secondly, the possibility of interaction between problem drinking and confounders – i.e. the possibility that the association is different for different levels of other risk factors — was explicitly considered and tested.

Thirdly, while most research on risk factors for TB (apart from the studies showing the effect of HIV infection) has been conducted in industrialized countries with a low prevalence of the disease [30], this study refers to a middle-income country with high TB burden. Its results thus contribute to the knowledge of factors affecting the development of TB in contexts in which the prevalence of TB is on the rise and scientific evidence to inform effective TB control policies is especially needed.

From a methodological point of view, the contribution of this study is the use of a PS matching procedure to deal with the potentially large bias introduced by traditional multivariate modelling in case of rare outcomes and multiple confounders, and the adaptation of the basic ideas of sensitivity analysis proposed by Ichino *et al.* to the case of estimation of prevalence odds ratios in the context of complex surveys.

The major limitations of this study derive from its cross-sectional design and the consequent use of measures of lifetime prevalence instead of incidence to infer causality. The intrinsic lack of temporal information attached to these measures, together with the unstable nature of problem drinking — characterised by multiple onsets followed by remission periods — did not allow us to establish the relative timing of exposure and outcome. This leaves room for a partially reverse causal interpretation, in which the observed association between the (lifetime) prevalence of TB and problem drinking is due to the overlapping of two different mechanisms: the effect of drinking in increasing the risk of TB infection and progression to disease (direct effect) and the effect of TB disease on problem drinking (reverse effect). As a consequence, our result would be an overestimation of the effect of problem drinking on TB. However, it is unlikely that the strength of the reverse effect (possibly mediated by psychological and social changes consequent to the onset of the disease, but for which no evidence could be found in literature) could exceed the direct effect, for which biological and sociological plausibility exists [1].

Using prevalence to estimate a causal effect is also a likely source of bias in our results. Even in case the assumption of a population in steady state holds, at least approximately, by using POR for inference we are incorporating in our measure of effect both a true causal parameter (i.e. the ratio between the incidence rate of TB among exposed and non-exposed) and a bias factor depending on the relative duration of the disease among those groups [31]. However, given that problem drinkers are likely to die earlier than non-problem drinkers (both because the exposure worsens the course of TB, and for reasons unrelated to TB [32]), it is likely that the results reported above represent an underestimation of the true causal effect of problem drinking on TB disease risk.

Low reliability of the self-report measures used in the SADHS for both the outcome and the exposure of interest could also have biased the results. On the plausible hypothesis that measurement error in the exposure (CAGE scores) is unrelated to TB status, it is likely — assuming that the error is also independent of the value of all confounders and the misclassification is not so severe that the estimate crosses to the opposite side of the null — that the observed POR has the same sign as the true association but reduced magnitude [33,34]. In this case more precise measurements would strengthen the result of our analysis rather than invalidate them.

By contrast, it is possible that misclassification of TB might be differential if problem drinking is associated with more frequent investigations for TB, which people might report as *having been told they had TB*. This lower specificity of the measurement of TB among drinkers than among non-drinkers could have introduced bias away for the null in the estimated POR, by generating more false positives (i.e. subject incorrectly classified as having had TB) among drinkers than among non-drinkers.

Low reliability is also likely to have affected the measurement of confounders (e.g. micronutrient deficiency, that, moreover, is reported in the dataset with no missing values, which could indicate some form of undocumented imputation). The direction of the bias in these cases is less predictable. However, due to robustness of the PS analysis in respect of misspecification of the models [18,35-37], we would expect only a modest amount of residual confounding for this reason.

Finally, even though suboptimal response rates as those observed in the SADHS do not automatically reflect in selection bias, especially in analytical studies [38], we cannot exclude the possibility that differences between respondents’ and non respondents’ drinking behaviour and TB status — untestable with the available data — could have affected our estimates of the association between problem drinking and TB

## Conclusion

In a cross-sectional national survey of the adult population of a middle-income country with a high TB burden, problem drinking was associated with a two-fold increase in the odds of past TB diagnosis after controlling for a large number of socio-economic and biological confounders. The estimated prevalence odds ratio was robust with respect to model misspecification and overadjustment for factors possibly lying in the causal pathway between exposure and outcome. Sub-population analyses did not offer evidence for sizable interaction between problem drinking and the considered confounders.

Within the limitations of a cross-sectional study design with self-reported TB status, these results confirm previous evidence from longitudinal research supporting the existence of a causal link between problem drinking and TB disease, and suggest that the observed higher prevalence of TB among problem drinkers cannot be attributed to the confounding effect of the uneven distribution of other risk factors.

## Competing interests

Both authors declare that they have no competing interests.

## Authors’ contributions

RE was involved in conception of the study and an early analysis. AC conceived the re-analyses and both authors were involved with data interpretation and critical revisions of the paper. Both authors read and approved the final manuscript.

## Pre-publication history

The pre-publication history for this paper can be accessed here:

http://www.biomedcentral.com/1471-2458/13/871/prepub

## Supplementary Material

Additional file 1 **Statistical analyses and further results.** Methodological details on statistical analyses. Further results. Click here for file
