# Supplementary material for: Problem drinking as a risk factor for tuberculosis: a propensity score matched analysis of a national survey
Source: BMC Public Health. 2013 Sep 22;13:871. doi: 10.1186/1471-2458-13-871 (PMC3852702; doi:10.1186/1471-2458-13-871)
Supplement: Additional file 1 — Statistical analyses and further results. Methodological details on statistical analyses. Further results. [file 1471-2458-13-871-S1.pdf]

Problem drinking as a risk factor for tuberculosis: a propensity score matched analysis of a national survey

Additional file 1: Statistical analyses and further results

Annibale Cois*[1] and Rodney Ehrlich[1]

[1]School of Public Health and Family Medicine, University of Cape Town, Anzio Road, Observatory 7925, Cape Town, South Africa

Email: Annibale Cois*- Annibale.Cois@uct.ac.za; Rodney Ehrlich - Rodney.Ehrlich@uct.ac.za;

*Corresponding author

Estimation of propensity scores

A probit model was used to estimate the propensity score (PS) including all potential confounders. As previously done by other authors (see, for example Zanutto *et Al.* [1] and Rubin [2]) we introduced population strata and sampling weights (categorised in quintiles) as further covariates. Population strata, in fact, carry information on place of residence which are not included in the other covariates and it is reasonable that an association could exist with both TB status and problem drinking, while is quite improbable that problem drinking would affect (in causal terms) population strata. Similarly, sampling weights are adjusted to take into account heterogeneous response rates across geographical and racial groups [3]. Thus, they might indirectly incorporate information on subjects' characteristics not captured by other covariates and be associated with both the outcome and the exposure.

The model used for the estimation of the PS in Stata® was, therefore (reference category for each variable omitted):

probit	PROBLEM	*Problem drinking*
	SEX	*Gender*
	AGE1 AGE2 AGE3 AGE4 AGE5	*Age categories*
	SMOK1 SMOK2	*Smoking status*
	BMI1 BMI2 BMI3	*BMI class*
	EDU1 EDU2 EDU3	*Education*
	WQ2 WQ3 WQ4 WQ5	*Wealth quintile*
	COL WHI ASI OTH	*Race*
	MICRO1 MICRO2 MICRO3	*Micronutrient deficiency*

OSMOK	*Occupational exposure to smoke*
DIAB	*Diabetes*
CROWD	*Overcrowding*
MEDAID	*Medical insurance*
RESID	*Residence*
SMFUEL	*Domestic exposure to smoke*
SWC1 SWC2 SWC3 SWC4	*Quintile of sampling weights*
STC2 ... STC18	*Sampling strata*

We also considered the inclusion of higher order and interaction terms, but the satisfactory results of the model above (see below) made it unnecessary to include these further elements.

Matching procedure and covariate balance assessment

To take advantage of the relatively large number of moderate drinkers/abstainers (unexposed) compared to problem drinkers ($>$ 5 unexposed per problem drinker) and to maximize power, a 1:4 matching ratio was chosen. Nearest neighbour matching with replacement was performed on the odds of PS (see below) with the user-written Stata® command *psmatch2* [4]. A caliper was used to limit the risk of mismatching and the 24 problem drinkers with PS higher than the largest PS among the unexposed were excluded from the analyses (restriction to the common support area). The size of the caliper was chosen according to the prevalent literature, and, in any case, the analyses were robust in respect of changes in the value of this parameter.

We used the user-written Stata® command *pstest* [4] to calculate (1) pre-and post-matching standardised percentage of bias for all observed covariates (Figure 1 in the article); (2) t-tests for equality of means of covariates across samples (all results not statistically significant after matching, with p-values $>$ 0.40).

The results of the procedure were satisfactory according to common criteria reported in literature [5], supporting the assumption that the proposed model achieved a good balance between problem drinkers and moderate drinkers/abstainers across all measured potential confounders.

Sub-population analysis for heterogeneity of effect sizes

The possibility of interaction between confounders and problem drinking in the association with TB was assessed repeating the whole procedure (including the estimation of propensity score) for selected sub-populations. To compare the POR between these sub-population, we estimated the 95% confidence intervals of their ratio using the procedure suggested by Altman and Bland [6].The results for the selected sub population are reported in Additional Figure 1.

Additional Figure 1: Ratio between POR in selected subpopulations and 95% confidence interval

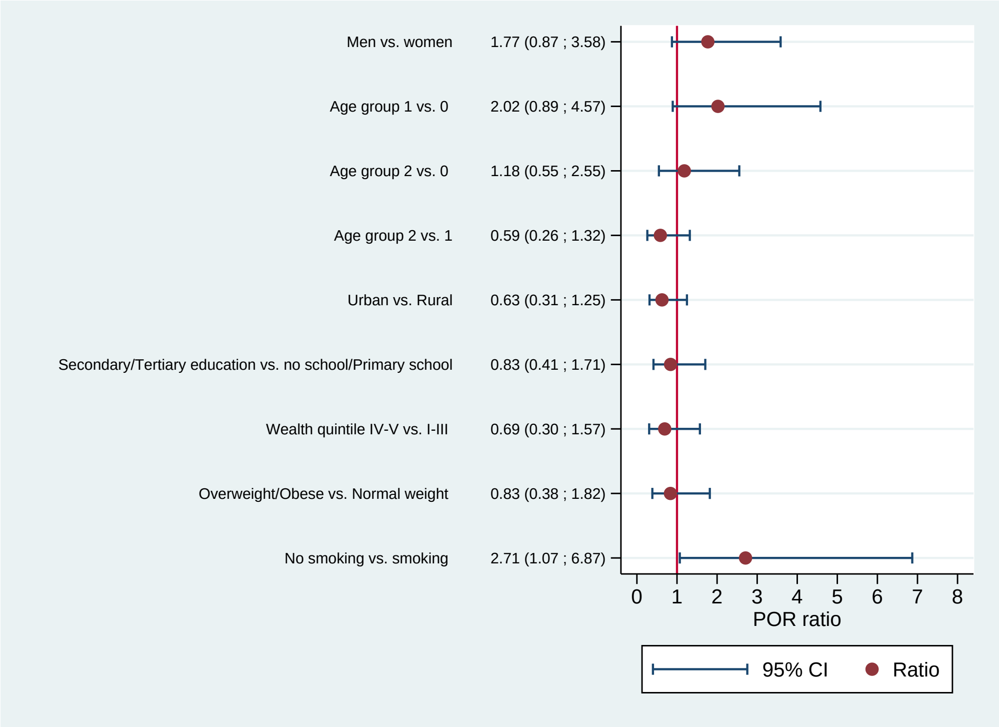

Sampling weights

Sampling weight were incorporated in the estimation of the POR applying sampling weights of problem drinkers also to the corresponding matched unexposed individuals [7].

Following the approach of Zanutto [8], we did not consider directly sampling weights in the estimation of PS but instead performed the matching procedure on the odds of the PS, which offers consistent results in samples drawn with unequal probabilities [9].

Confidence intervals

Calculation of valid confidence intervals in the context of PS analysis is a controversial subject in the literature, and no general analytical expression exists to calculate the variance of the effect sizes estimates. Several approximations are in use in special cases, but no one is applicable to our analytical design in which the estimated parameter is the *ratio* between two odds — instead of the *difference* between means or proportion most commonly considered— and in which a complex survey design is also involved. [10, 11]

We therefore calculate approximate confidence intervals through bootstrapping [12]. This method, despite having been shown inconsistent in some cases [13], is widely applied in the context of PS analysis (see e.g. Austin [14], Bryson [15] or Larsen [16]).

In the estimation of the 95% confidence intervals the whole procedure of POR estimation — including the calculation of the PS — was replicated 750 times taking into account the sampling scheme though the Stata® command *svy bootstrap*. The number of replications was chosen both in reference of the mainstream literature on bootstrapping [10]) and observing the empirical distribution of the estimated confidence intervals (Additional Figure 2) for different number of replication, which shows a reasonable convergence (changes in the third decimal digit) for values greater than 750.

Sensitivity analysis in respect to unmeasured confounders

The method we used to assess the sensitivity of the POR estimate to the presence of unobserved covariates associated with both the exposure and the outcome is an adaptation of the procedure proposed by Ichino *et Al.* [17] and implemented in the user-written Stata® command *sensatt* [18]. We modified the original procedure implemented in *sensatt* in two ways:

1. We used as a measure of effect not the average treatment effect (ATE) but rather the POR;
2. We represented graphically the results of 1000 replications of the procedure (with different values for the prevalence of U among exposed/unexposed and subjects with/without the outcome) with a contour plot in which the axes were the adjusted odds ratios of the association of U with the exposure and the outcome (Figure 4 on the article). This allowed us to visually identify lower bounds for the strength of the association that potential extra confounders would have to have with the outcome and the exposure to offset the observed POR or to reduce its value below any specific threshold.

Results from logistic regression

By way of comparison with traditional outcome modelling, we calculate the POR of TB disease among problems drinkers vs. moderate drinkers/abstainers with logistic regression, including in the model the same set of covariates used for the estimation of the PS (excluding population strata and sampling weights). The estimated POR is in this case 1.69 (95% CI: 1.07 to 2.67), which is lower than the results of the PS matching analysis.

References

1. Zanutto E, Lu B, Hornik R: **Using Propensity Score Subclassification for Multiple Treatment Doses to Evaluate a National Antidrug Media Campaign**. *J Educ Behav Stat* 2005, **30**:59–73.
2. Rubin DB: **Using propensity scores to help design observational studies: application to the tobacco litigation**. *Health Serv Outcomes Res Methodol* 2001, **2**:169–188,.

3. Department of Health, Medical Research Council, OrcMacro: *South Africa Demographic and Health Survey 2003*. Pretoria: Department of Health 2003.

4. Leuven E, Sianesi B: **PSMATCH2: Stata module to perform full Mahalanobis and propensity score matching, common support graphing, and covariate imbalance testing.** *Statistical Software Components S432001 (revised 19 Jul 2012). Newton, MA: College Department of Economics* 2003, [http://ideas.repec.org/c/boc/bocode/s432001.html].

5. Austin PC: **A critical appraisal of propensity-score matching in the medical literature between 1996 and 2003.** *Statistics in medicine* 2008, **27**(12):2037–49.

6. Altman DG, Bland JM: **Interaction revisited: the difference between two estimates.** *BMJ* 2003, **326**(7382):219.

7. Frolich M, Heshmati A, Lechner M: **A Microeconometric Evaluation of Rehabilitation of Long-term Sickness in Sweden**. *J Appl Econom* 2004, **19**:375–396.

8. Zanutto E: **A Comparison of Propensity Score and Linear Regression Analysis of Complex Survey Data**. *J Data Sci* 2006, **4**:67–91.

9. Smith JA, Todd PE: **Does matching overcome LaLonde's critique of nonexperimental estimators?** *J Econometrics* 2005, **125**(1–2):305–353.

10. Hill J, Reiter JP: **Interval estimation for treatment effects using propensity score matching.** *Stat Med* 2006, **25**(13):2230–56.

11. Ho DE, Imai K, King G, Stuart EA: **Matching as Nonparametric Preprocessing for Reducing Model Dependence in Parametric Causal Inference**. *Political Analysis* 2007, **15**(3):199–236.

12. DiCiccio TJ, Efron B: **Bootstrap Confidence Intervals**. *Stat Sci* 1996, **11**(3):pp. 189–212.

13. Abadie A, Imbens GW: **On the failure of bootstrap for matching estimators**. *Econometrica* 2008, **76**(6):1537–1557.

14. Austin PC: **A Tutorial and Case Study in Propensity Score Analysis: An Application to Estimating the Effect of In-Hospital Smoking Cessation Counseling on Mortality.** *Multivar Behavi Res* 2011, **46**:119–151.

15. Bryson A: **The Union Membership Wage Premium: An Analysis Using Propensity Score Matching,**. Tech. rep., Centre for Economic Performance London School of Economics and Political Science, London 2002.

16. Larsen MD: **An analysis of survey data on smoking using proensity scores**. *Sankhya Ser B* 1999, **61**(pt. 1):91–105.

17. Ichino A, Mealli F, Nannicini T: *From Temporary Help Jobs to Permanent Employment: What Can We Learn from Matching Estimators and their Sensitivity?* Bonn: IZA 2006.

18. Nannicini T: *SENSATT: Stata module to compute sensitivity for matching estimators.* No. S456747 in Statistical Software Components, Boston: Boston College Department of Economics 2006.
